# Similarity based functionalization for enumeration of synthetically plausible chemical libraries surrounding a target†

**DOI:** 10.1039/d4sc00523f

**Published:** 2024-05-24

**Authors:** Karthik Sankaranarayanan, Klavs F. Jensen

**Affiliations:** a Department of Agriculture and Biological Engineering, Purdue University West Lafayette Indiana 47907 USA ksankara@purdue.edu; b Department of Chemical Engineering, Massachusetts Institute of Technology 77 Massachusetts Avenue Cambridge Massachusetts 02139 USA kfjensen@mit.edu

## Abstract

Functionalization of lead compounds to create analogs is a challenging step in discovering new molecules with desired properties and it is conducted throughout the chemical industry, including pharmaceuticals and agrochemicals. The process can be time-consuming and expensive, requiring expert intuition and experience. To help address synthesis planning challenges in late-stage functionalization, we have developed a molecular similarity approach that proposes single-step functionalization reactions based on analogy to precedent reactions. The developed approach mimics reaction strategies and suggests co-reactants defined implicitly by a corpus of known reactions. Using *ca.* 348 k reactions from the patent literature as a knowledge base, the recorded products or close analogs are among the top 20 proposed products in 74% of ∼44 k test reactions. The combinatorial growth inherent in recursive applications of the tool allows the enumeration of chemical libraries surrounding a target compound of interest. Moreover, each step of the resulting library synthesis leverages common chemical transformations reported in the literature accessible to most chemists.

## Abstract 2

Given a late-stage reactive intermediate as input, the algorithm, enabled by a large corpus of organic reactions, identifies different reactive sites within the molecule and proposes suitable transformations for diversification at every site.

## Graphical abstract

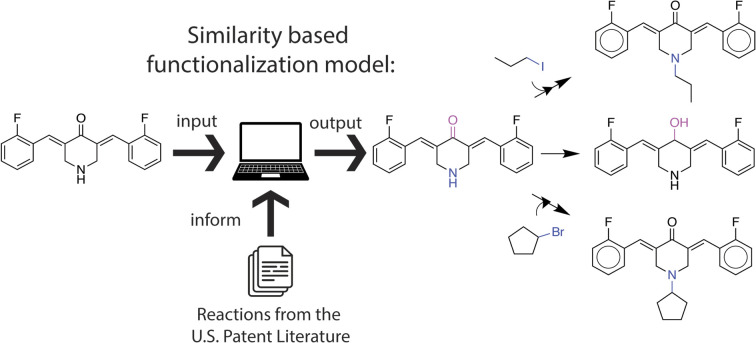

## 1. Introduction

Late-stage diversification (LSD) is a synthetic strategy in which a complex reactive intermediate in the final stages of synthesis is diversified to generate new analogs of a lead structure without resorting to *de novo* synthesis.^1^ Reactive sites on the intermediate, including functional groups and C–H bonds, are points of potential diversification for analog generation. LSD could facilitate the development of structure–activity relationships (SAR), optimization of potency, safety, absorption–distribution–metabolism–excretion properties, and improvement of physical properties such as solubility.^2^ Notwithstanding impressive advances in late-stage diversification experimental strategies, there remains a need for the development of computational tools for planning late-stage synthesis strategies for generating a large virtual library of analogs of a target compound of interest.

Generative models use recent advances in digitization of large datasets and deep learning to produce candidate analogs.^3^ However, many common generative models do not consider synthetic feasibility of the analogs generated.^4^ This inability of generative models to consider synthesizability constrains their use in drug discovery. As a result, there has been growing interest in the generation of synthesizable molecules.^5,6^ For example, a recent model developed by Gao *et al.* generates molecules in a bottom-up manner, starting from available building blocks and building up progressively to more complex molecules.^5^ This generation task is conditioned on an embedding for a target molecule. If the target molecule is reachable using the curated template set and building blocks, the final molecule matches the input target molecule or is a close analog. This approach relies on a manually curated set of 91 reaction rules in organic chemistry and does not explicitly consider late-stage diversification of complex intermediates. A second representative approach developed by Sauer *et al.* generates molecules using a fragmentation and replacement scheme.^6^ Here, molecules are represented as fragments, and new molecules are constructed by replacing some fragments of the input compound with others from a large library of fragments. To improve synthesizability of generated molecules, this approach relies on a manually curated set of 6 reaction rules in organic chemistry. There remains a need for computer-aided (CASP) synthesis planning models that employ a wide range of organic transformations to diversify late-stage intermediates.

Reaction templates, subgraph patterns that describe changes in connectivity between the product molecule and its corresponding reactants, have been used to enumerate a large library of analogs.^7–9^ For example, a recent approach developed by Levin *et al.* performs combinatorial enumeration by querying a large database of building blocks using structural patterns present in reaction templates within a synthetic route.^7^ A second representative approach developed by Dolfus *et al.* starts with a retrosynthetic analysis for the input compound to yield a synthetic plan.^8^ Then, it performs combinatorial enumeration by querying a database of building blocks using substructure patterns present in reaction templates of the synthesis plan. Both approaches use algorithmically extracted reaction templates to propose and assess the feasibility of enumeration reactions. However, these templates are localized to the reactive site and do not consider contributions to reactivity by distant functional groups away from the reactive site. In some cases, functional groups present far from the reactive site could contribute beneficially to the proposed reaction, *e.g.*, through some enabling context or activation. In other cases, these distant functional groups could contribute detrimentally to the proposed transformation, *e.g.*, by creating a competing reaction channel. As discussed by the Levin and co-authors, the use of substructure matching is not a sufficient criterion for determining experimental substrate compatibility, but a useful starting point. Using reaction templates for enumeration also constrains the modification to a single reactive site and a single reaction chemistry. Algorithms that can identify multiple reactive sites within a molecule and employ suitable reactive chemistries would increase the diversity of generated analogs.

Information retrieval using chemical similarity is an effective and versatile strategy for performing retrosynthesis.^10,11^ RDKit enables facile setup and testing of similarity-based algorithms because of its implementation of Morgan circular fingerprints and similarity metrics (*e.g.*, Tanimoto, Dice, and Tversky). As new data is generated in the literature, these algorithms do not require retraining, making them simpler to maintain compared to their machine learning counterparts. Finally, similarity-based approaches are intuitive to chemists, who are frequently the end users of computational chemistry tools designed for drug discovery. These approaches identify literature precedents that are chemically similar to the proposed transformations, in a fashion similar to a chemist performing a search in the literature or in one of the chemical reaction databases (*e.g.*, Reaxys, SciFinder, and Science of Synthesis). In contrast, suggestions proposed by their machine learning counterparts are a generalization of literature precedents. Herein, we use chemical similarity for proposing reactions to generate analogs starting from a late-stage reactive intermediate.

To propose diversification transformations by generalizing reactions in a database, the task of reaction template extraction and application plays a central role. *In lieu* of developing a new tool, we propose leveraging similar template extraction and application tools designed for retrosynthesis, a well-studied problem. For example, RDChiral is a tool designed to handle templates for retrosynthesis.^12^ Because of the strong interest in retrosynthesis, this tool can handle stereochemistry and double bond configurations. To successfully leverage RDChiral for late-stage diversification, two key challenges need to be solved. First, late-stage diversification needs to be formulated appropriately as the inverse of retrosynthesis. Second, many diversification transformations add new structural components to the input molecule. Co-reactants or building blocks have to be implicitly included in the reaction templates in this problem formulation strategy. Herein, we propose a problem formulation that enables us to leverage RDChiral. We further modify RDChiral to implicitly incorporate any co-reactants or building blocks into the reaction templates.

## 2. Methods

Our functionalization strategy starts by asking the question: How have chemically similar molecules been transformed previously using common chemistries reported in the patent literature? By ensuring chemical similarity between proposed and precedent reaction molecules, this approach intends to propose transformations that have literature precedence, and therefore more likely to be experimentally feasible. Our methods section formalizes this workflow, and it is organized into three sub-sections:

(1) The source of data for our experiments.

(2) The algorithm for enumeration and analog generation.

(3) The metric employed to quantitatively evaluate algorithm performance.

### 2.1. Dataset

As a source of data, we used reactions from USPTO granted patents, originally collected by Lowe^13^ and further modified by Jin *et al.*^14^ Previously, Schneider *et al.* randomly sampled *ca.* 50 k reactions from the patent literature, and Table 1 describes the distribution of reaction types in this subset.^15^ As a consequence of their random sampling, this should also approximate the true distribution in the full USPTO literature. Our data-driven approach intends to offer insights that can be achieved by a trained chemist familiar with these reaction types. Because these reactions are commonly employed in synthetic chemistry and are familiar, the proposed suggestions would be amenable to rapid and economic experimental implementation.

**Table 1:** Distribution of reaction classes in the USPTO-50 k reaction dataset, adapted from Schneider *et al.*^15^

Description	Fraction of the dataset (%)
Heteroatom alkylation and arylation	30.3
Acylation and related processes	23.8
C–C bond formation	11.3
Heterocycle formation	1.8
Protections	1.3
Deprotections	16.5
Reductions	9.2
Oxidations	1.6
Functional group interconversion (FGI)	3.7
Functional group addition (FGA)	0.5

The dataset, published by Jin *et al.*,^14^ has *ca.* 480 k reactions. We additionally processed this dataset so that each reaction example had a single reactant. For a reaction with multiple reactants, we constructed a corresponding pseudo reaction that contained the most complex reactant and the original products; the other less complex reactants were removed from the reaction (henceforth, these removed reactants are referred to as ‘coreactants’). 88% of the reactions in the database had coreactants that were listed as commercially available in a buyable database or did not require any coreactants (see ESI†). The construction of these pseudo reactions was necessary to leverage RDChiral, a template extraction and application tool for retrosynthesis.^12^ To help identify the most complex reactants in a reaction, molecular complexity was evaluated using the SCScore model previously developed by Coley *et al.*^16^ The stronger emphasis on complex reactants in the processed dataset is because of our goal of developing a tool for late-stage functionalization, where our input molecules also tend to be complex. We emphasize that this was simply a choice; therefore, utilizing other metrics of molecular complexity or parsing multi-reactant reactions into multiple single reactant reactions are valid alternatives to our approach.

RDChiral was used for template extraction and application.^12^ By design, this problem was formulated as an inverse of the retrosynthesis problem. The roles of reactants and products of the pseudo reaction were reversed during the retrosynthetic template extraction. That is to say, the reactants of the pseudo reaction were fed as products to RDChiral and *vice versa*. This allowed us to take advantage of the techniques that have been developed and refined for retrosynthetic template extraction and application. As a quality control, we applied the extracted templates to the reactants in the dataset and we ensured that we were able to recover their corresponding products. Reactions that did not pass this quality control check were removed from the dataset; less than 3% of the total dataset were removed in this step (12 511 removed reactions of 447 757 total reactions).

Next, we canonicalized the reaction SMILES string and removed duplicate transformations. The dataset was split randomly into training (80%), validation (10%), and test splits (10%) using the reactant SMILES string. We split the dataset by reactant because this ensures the algorithm has neither seen the input compound nor the associated reaction when it attempts to predict the recorded product of the test set.

### 2.2. Algorithm for enumeration and analog generation

**Fig. 1:**
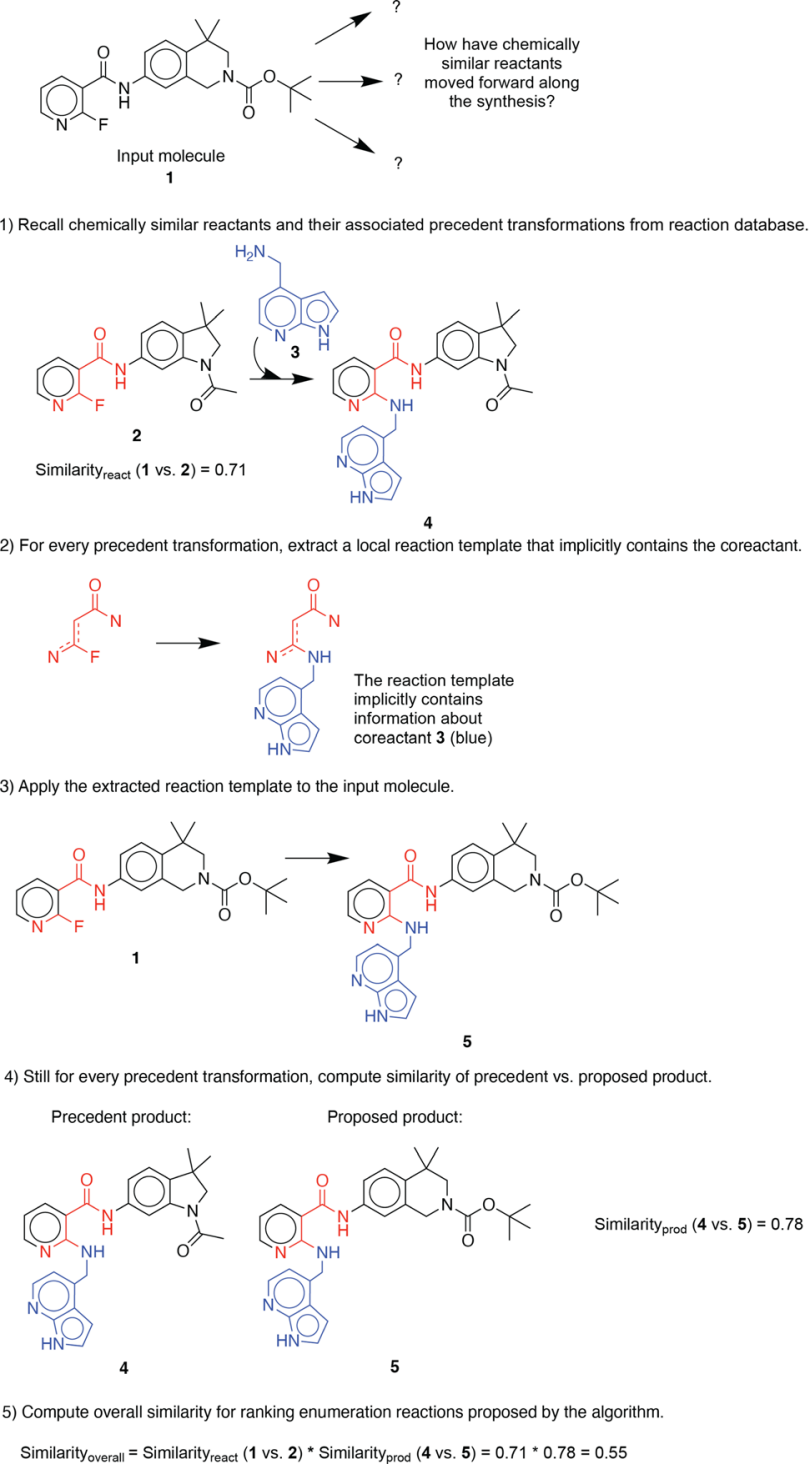
An example illustration of the stepwise procedure for enumerating possible single-step functionalization reactions.

For illustration purposes, we use an exemplary molecule from the dataset previously unseen by the algorithm. First, the molecular similarity is utilized to propose one-step reactions based on analogy to precedent reactants in a reaction database (in this example, similarity_reactant_ = 0.71) (Fig. 1). Second, a generalized forward template, which implicitly contains necessary co-reactants, is extracted from the precedent reaction. Third, the template is applied to the input molecule to generate its corresponding product. Then, the chemical similarity of the proposed and precedent products is calculated (in this example, similarity_product_ = 0.78). Finally, proposed reactions are scored and ranked by overall molecular similarity, defined as similarity_reactant_ × similarity_product_ (in this example, similarity_overall_ = 0.55), to the precedent reaction. This approach was adapted from a previous investigation on utilizing chemical similarity for proposing retrosynthetic suggestions.^10^ A more detailed description of its implementation can be found in the ESI.†

Similarity computations require a fingerprinting technique and a similarity metric. We tested a range of Morgan fingerprint settings and similarity metrics in a combinatorial fashion. Specifically, we tested Morgan fingerprints of radii = {2,3} and with/without features, as implemented by RDKit. Similarity, we tested the Dice, Tanimoto, and Tversky similarity metrics. For illustration purposes, here we compute molecular similarity using Morgan fingerprint (radius = 2, using features) and Tanimoto similarity metric for the reference molecule (6) from the test set previously unseen by the algorithm (Table 2). Changes at an atom level result in a measurable change in the similarity score. As a result, similarity provides an indication of the presence or absence of functional groups in the target compound *vs.* the precedent reactant. The approach hypothesizes that reactions associated with chemically similar compounds (7)–(10) are likely applicable to compound (6). Recorded reactions associated with compounds (6)–(10) are available in the ESI, Fig. S1.†

**Table 2:** Similarity score calculation. Example similarity score calculation using *Morgan2Feat* fingerprint and the Tanimoto metric. Colors indicate atom-level contributions to the overall similarity (green: increases similarity score, red: decreases similarity score)

Target compound (test data set)	Precedent reactants that appear in the training set and their corresponding similarity scores
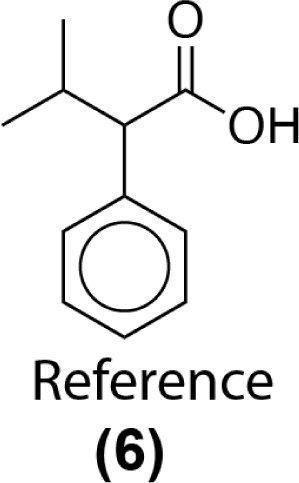	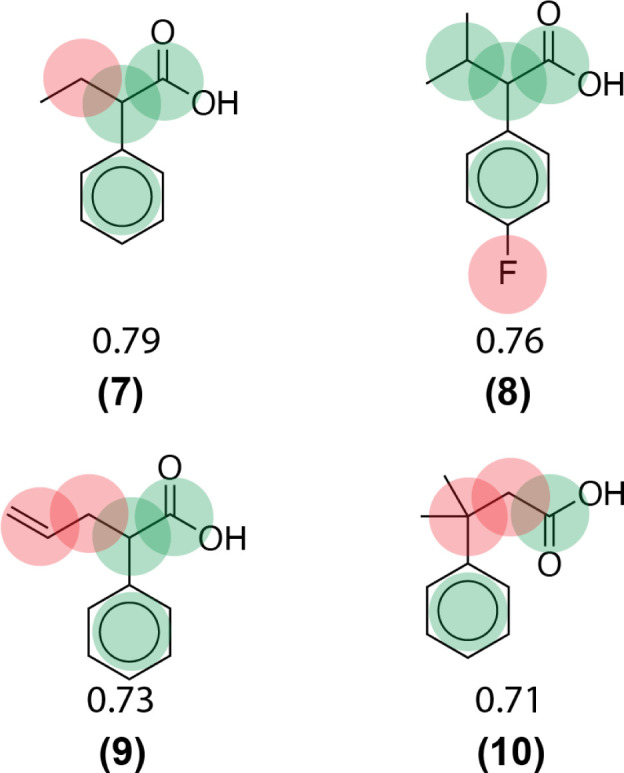

**Fig. 2:**
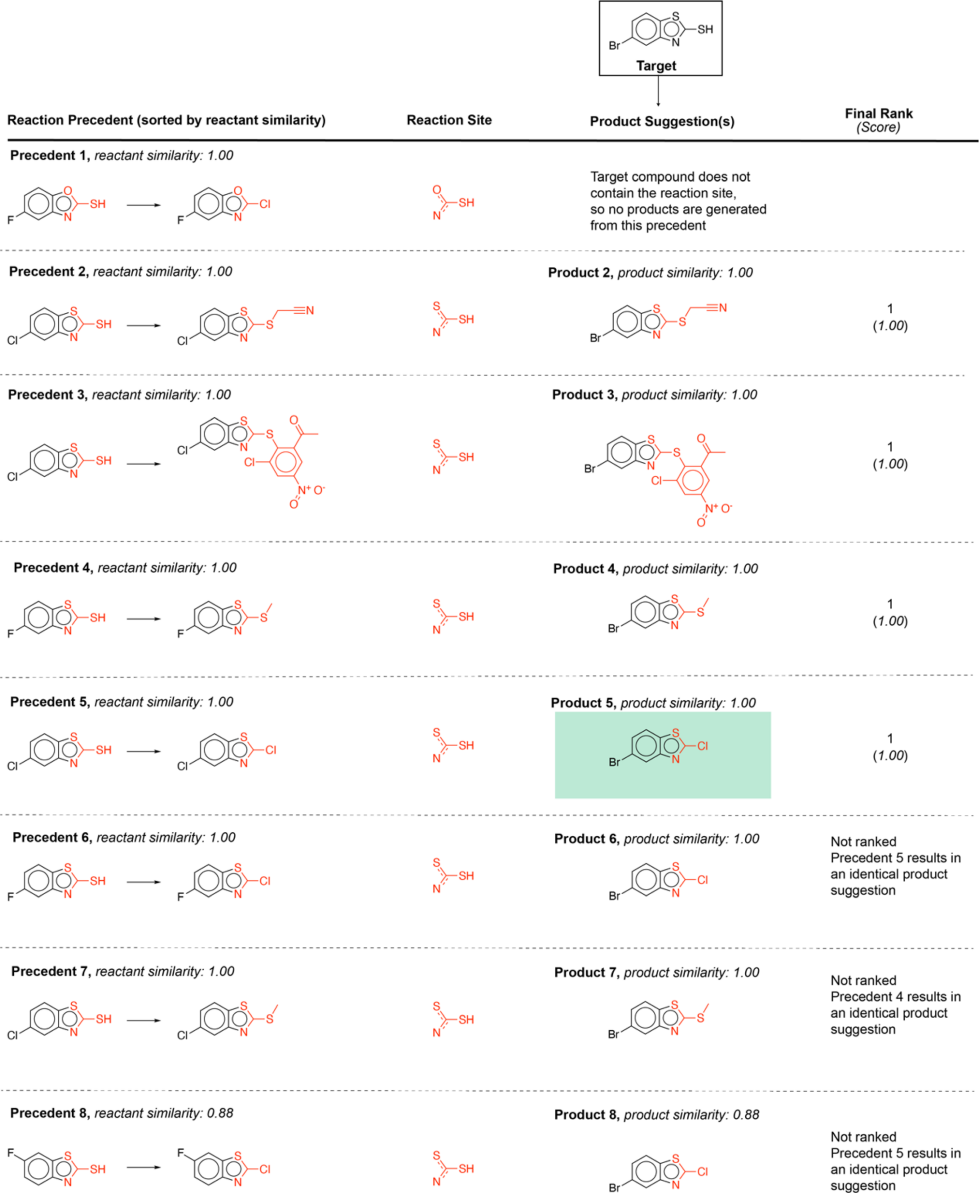
Example forward reaction predictions for a target compound that appears in the test data. The precedent reaction sites are highlighted in red. The recorded product for this target compound is highlighted inside a green box.

Using this computed similarity metric, the algorithm recalls reaction precedents in the order of decreasing reaction similarity (for illustration purposes, only top-8 reaction precedents are shown in Fig. 2). Then, the precedent reaction site is extracted and matched against the target compound. Of the precedent reactions with the most similar reactants, not all involve a reaction site that matches the target compound and thus not all produce candidate products. Duplicates in the candidate product list-such as products 6, 7, and 8 (Fig. 2)- were removed, while retaining only the highest score when there are multiple entries. The recorded product for this target compound is recovered and predicted with rank 1; however, all the top 4 suggestions are chemically reasonable.

### 2.3. Evaluation procedure

**Fig. 3:**
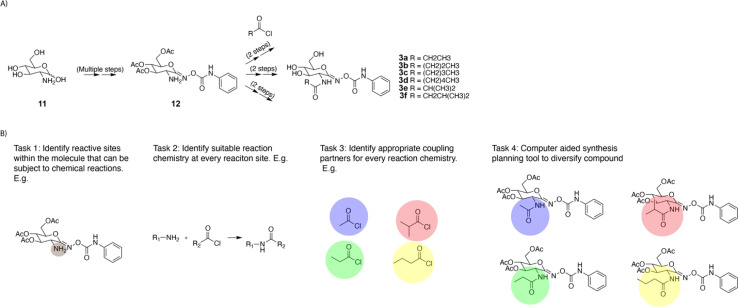
Graphical summary of the evaluation procedure. (A) Example functionalization example from the literature.^17^ (B) Illustrations of the four tasks involved in the evaluation procedure.

Forward enumeration implies finding single-step chemical transformations that change the chemical structure of the desired reactive intermediate. Further, the proposed reactions should have a high likelihood of success in the forward direction. In an exemplary divergent synthesis strategy previously implemented by Stubbs *et al.*,^17^ the complex intermediate 12 containing an amine was treated with different acyl chlorides to synthesize a library of analogous compounds (Fig. 3A). The single step enumeration algorithm should take compound 12 as its input, and it should propose reactions to generate a library of analogous molecules. To facilitate the design of the algorithm, this large task can be broken down into four smaller tasks (Fig. 3B). First, the algorithm needs to identify suitable reaction site(s). Second at each potential reaction site, it should identify suitable reaction chemistry. Third for every reaction chemistry, it must select a suitable co-reactant. Finally using all outputs from tasks 1–3, the algorithm needs to generate a library of analogous products. Algorithm performance on these four tasks will be measured quantitatively. While the evaluation procedure is formalized in the next paragraph, a more detailed description of its implementation can be found in the ESI.†

To evaluate our algorithm, we use the following success criterion: given the reactant of reactions in the United States patent literature, the program recovers and ranks highly close analogs of the recorded product without having seen that reaction previously. To determine whether the proposed product is an analog of the recorded product, two similarity computations were performed. First, the similarity of the recorded reactant to the recorded product was computed using their Morgan fingerprints and Tanimoto similarity (defined as *s*_1_). Second, the similarity of the algorithm's proposed product to the recorded product was computed using their Morgan fingerprints and Tanimoto similarity (defined as *s*_2_). If *s*_2_ > *s*_1_, the proposed product is considered an analog, and the most similar analog, with the highest *s*_2_ similarity score, was used for rank determination. Performance is measured using top-*N* accuracy for *N* = {1,3,4,10,20,50}; this is defined as the fraction of test set examples where close analogs of the recorded product are suggested by the algorithm with rank ≤ N. This evaluation metric would provide quantitative support for the algorithm's capability to propose feasible transformations that alter the chemical structure of the desired compound. It checks to see if, at a minimum, the known reaction strategy published in the patent literature is successfully recovered by the algorithm.

## 3. Results

Different combinations of fingerprint settings and similarity metrics were evaluated using the validation dataset (Fig. S2†). The top-*N* accuracy is not a strong function of the settings tested. As a result, Morgan fingerprint (with radius = 2, with features) and Tanimoto similarity metric are used in this algorithm. The test set top-*N* accuracy for the final algorithm is shown in Table 3. Given reactant molecules from the test set as input, the algorithm can recover close analogs of the recorded product with the top 1, top 5, top 20 suggestions 26%, 49%, 74% of the time, respectively. Five randomly selected examples from the test set are presented in Fig. S3–S7.† In Fig. S4–S6,† the algorithm recovers the recorded product exactly. In Fig. S7,† it recovers a close analog of the recorded product. In Fig. S3,† the algorithm is not capable of capturing the reaction strategy to recover an analog of the recorded product.

**Table 3:** Model performance. Given the reactant of reactions in the United States patent literature, the program recovers and ranks highly the recorded products or close analogs without having seen that reaction previously. Randomly selected examples from the test set are shown in Fig. S3–S7

Top-*n*	Accuracy (%)
1	26
3	41
5	49
10	61
20	74
50	86

**Fig. 4:**
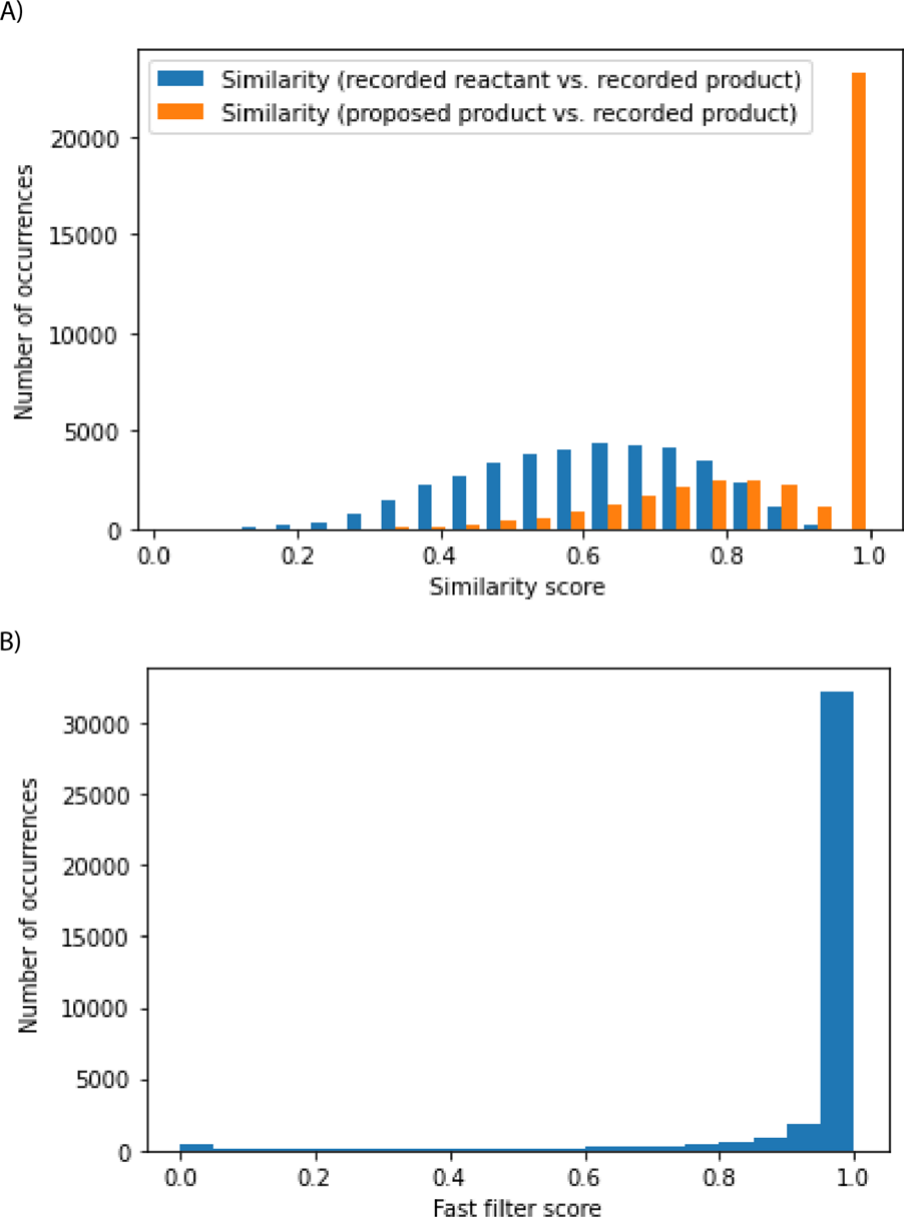
Analysis of reactions that meet the success criterion in the top-k accuracy analysis. (A) The similarity of recorded reactants to the recorded products was computed using Morgan fingerprints and Tanimoto similarity (in blue). The similarity of the algorithm's proposed products to the recorded products was computed using Morgan Fingerprints and Tanimoto similarity (in orange). (B) Most reactions considered successful have fast filter scores close to 1.

In the test set comprising 44 k reactions, roughly 90% of the cases were able to recover recorded products or close analogs, and these cases considered to be successful are further analyzed (Fig. 4). The algorithm can select reactions that shift the chemical distribution of this 90% of the test set away from the reactants towards the recorded products, without having seen the reaction and the reactant previously (Fig. 4A). These 90% of test set reactions considered successful are chemically sensible. The fast filter is a binary classifier that predicts whether reactions are feasible, and it is a deep neural network training on positive and generated negative examples from Reaxys.^18^ Model scores can range from 0 to 1, and they estimate the probability that the corresponding reactions can be implemented experimentally. Most reactions have fast filter scores close to 1, suggesting the promising nature of the reactions proposed by the algorithm (Fig. 4B). Likewise, a graph-convolutional neural network model trained by Coley and co-authors also predicted these reactions to be sensible (see ESI†).^19^ The fast-filter model has the advantage of speed and computational efficiency. On the other hand, the WLN has improved predictive capability at the expense of computational effort.

**Fig. 5:**
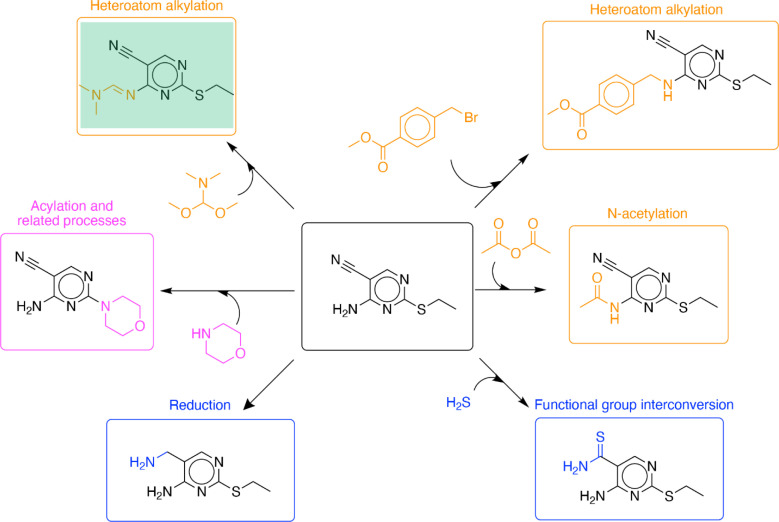
Example single-step functionalization reactions predicted for a target compound from the test set. The recorded product for this target was successfully recovered (highlighted in green box). Further, the algorithm proposes diverse functionalization reactions at different positions within the molecule (in different colors).

The algorithm can propose diverse functionalization reactions at different positions within the molecule. Such suggestions for a molecule found in the test set are shown in Fig. 5. The algorithm performance will be discussed in the context of the four tasks of single-step enumeration described previously in the methods section (‘C. Evaluation’). Given the target compound, it was able to identify multiple reactive sites within the compound (Fig. 3, task 1). The nitrile, amine, and sulfide functional groups were identified as potential reactive sites. Then for every reactive site, it identified suitable reaction chemistries (Fig. 3, task 2) and co-reactants (Fig. 3, task 3). Reactions proposed by the algorithm belong to diverse reaction classes including heteroatom alkylation, *N*-acetylation, functional group interconversion, reduction, and acylation and related processes.^20^ Finally, it moved the reaction forward by a single step to generate analogous molecules (Fig. 3, task 4). In addition to recovering the recorded product associated with the input compound, the algorithm proposes many sensible analogs. Using a knowledgebase of chemical transformations, the algorithm takes a holistic view of single step enumeration; it proposes a diverse set of analogs using a range of common chemistries reported in the patent literature.

**Fig. 6:**
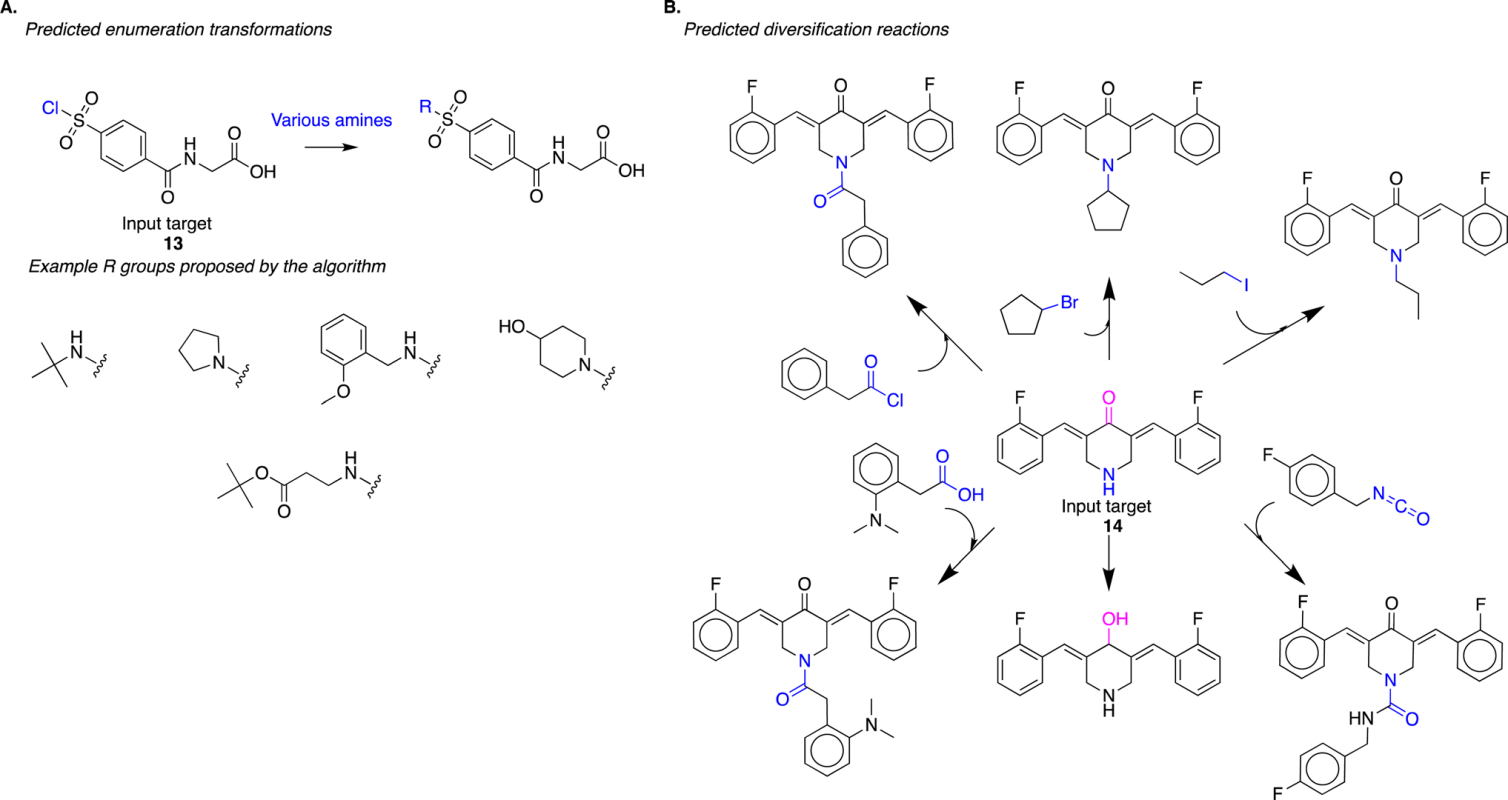
(A) The algorithm proposes the diversification of reactive intermediate 13. The strategy is similar to one experimentally implemented by Mann and co-authors (Fig. S8†); the algorithm proposes the treatment of 13 with various amines to yield the corresponding *N*-substituted-sulfonamides. (B) The algorithm predicts diversification of reactive intermediate 14. This proposed strategy modifies the input compound at multiple sites (in pink and blue) using different reaction chemistries.

The algorithm captures reaction strategies commonly employed by human experts for diversifying reactive intermediates for drug discovery. In a recent study, Mann and co-authors sought to discover inhibitors of the enzyme USP-5 through structure–activity relationship studies.^21^ To diversify the lead compound, they treated reactive intermediate 13 with various commercially available amines to obtain *N*-substituted-sulfonamides (exemplary reaction scheme reproduced in Fig. S8(A)†). Our approach identified a similar diversification strategy; it suggested the treatment of 13 with various amines to obtain *N*-substituted-sulfonamides (Fig. 6A). From the standpoint of the evaluation procedure outlined in Fig. 3, the algorithm identified the appropriate reaction site (task 1), reaction chemistry (task 2), and suitable co-reactants (task 3) to produce analogs (task 4). Here, the algorithm employed a single reaction chemistry and varied co-reactants to generate the different analogs. While this is an important strategy for diversification, we note that this task can be performed trivially using other computational approaches. For example, identifying couple partners with an amino functional group that will match a generalized reaction SMARTS pattern describing this transformation could accomplish this task just as efficiently.

The more challenging diversification planning, from a computational perspective, is identifying reactions where generalized SMARTS patterns might not overlap. This includes diversifying using different functional groups present in the input molecule. Similarly, diversification transformations with different reaction chemistries, *i.e.*, strategies where atoms and bonds that change during the reaction might be different, will not have identical SMARTS patterns. Lagisetty and co-authors diversify the reactive intermediate 14 at different sites and using different reaction chemistries (exemplary reaction scheme reproduced in Fig. S8(B)†);^22^ this strategy will have minimal reaction SMARTS overlap. Our approach identified a similar diversification strategy; it suggested diversifying 14 at different sites using different reaction chemistries (Fig. 6B). In this strategy, there is minimal reaction SMARTS overlap, presenting an example of diversification synthesis planning that cannot be trivially performed using more straightforward computational approaches. It requires intuitive knowledge about potential reaction sites within a molecule and potential reaction chemistries that could be employed at each of the identified sites. Our algorithm generalizes reaction information present in the USPTO reaction dataset to propose these new transformations.

The analogs generated by the algorithm can be further filtered using a molecular property prediction algorithm or a human expert with intuitive knowledge about the molecular features that might result in favorable properties. We note that while the algorithm is capable of generating candidate analogs that are chemically sensible and that can be achieved through known organic transformations, it is not designed to optimize the molecules towards any desired property. As a result, we highlight any overlap between published and predicted diversification strategies without a strong emphasis on an exact match for analogs generated.

**Fig. 7:**
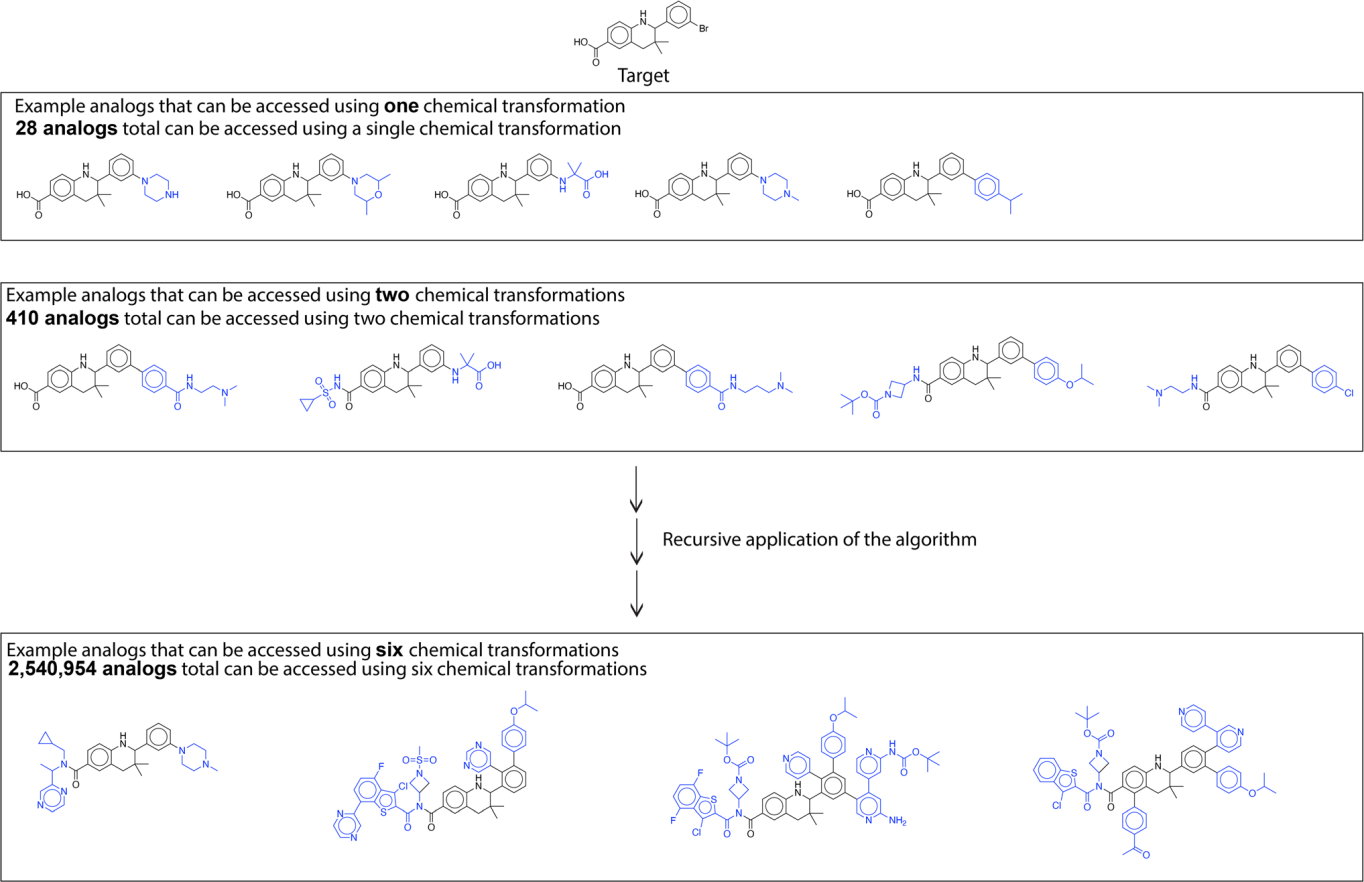
Library generation. Analogs are generated through recursive application of similarity-based enumeration.

A large library of analogs can be generated through recursive application of this similarity based single step enumeration algorithm (Fig. 7). At every iteration and for every input molecule, the number of analogs generated was limited for computational efficiency and to prevent rapid combinatorial growth. Consequently, we only consider the reactions corresponding to the top-100 reactants in the dataset most similar to the input molecule. This parameter can be set by the user after considering the availability of computational resources, desired feasibility of proposed solutions, and target number of generated analogous compounds. At every iteration, the fast filter was used for binary classification of reaction feasibility.^18^ The fast filter threshold was set at 0.5, and analogs generated by the algorithm using transformations below the threshold were not considered in subsequent iterations. By using parallel computing techniques, this single-step enumeration and subsequent fast filter validation workflow was performed efficiently within a reasonable time. The number of analogs generated every iteration and the time taken are available in Table S1.† These analogs can be further filtered using a property prediction algorithm to identify analogs with a desired property. As an example, we evaluate the library of analogs generated by the recursive application of similarity-based enumeration in Fig. 7 using the ‘QED’ property filter in the ESI.†

## 4. Discussion

Chemical innovation is pivotal to developing the next generation of products – including new pharmaceutical agents for patients, less expensive food and flavoring products for consumption, and efficacious agrochemicals that are also safer for farmers to handle. The establishment of structure–activity relationships of lead compounds is an important component of molecular discovery programs within these industries. Here, we have developed an algorithm to automate synthesis planning for these structure–activity relationship studies. It proposes chemical transformations to change the structure of the input molecule, often a late-stage reactive intermediate. To help chemists generate new ideas for diversifying a molecule, the algorithm identifies different reactive sites within a molecule and proposes suitable transformations for diversification at every site. Further, it can also be used recursively to generate a large virtual library of analogs for *in silico* screening using property prediction models.

This algorithm was developed by formulating this problem as the inverse of retrosynthesis, in which chemists work backwards from the desired product to determine the starting materials from which it is made. Computational retrosynthesis is a well-studied problem; the earliest publications on this topic were presented over 50 years ago.^23,24^ Both similarity searching and reaction template handling components of this study were repurposed after their initial use in organic retrosynthesis.^10,12^ As a result, our algorithm is able to effortlessly generalize reactions reported in the U.S. patent literature to propose new reactions for diversifying an input molecule. It is also well equipped to handle stereochemistry and double bond configurations because of its original use in retrosynthesis.^12^ This problem formulation strategy provided facile access to the use of mature computational tools developed for retrosynthesis to begin solving the challenge of late-stage diversification and synthesis planning for structure–activity relationship studies.

To improve the quality and utility of diversification suggestions, this tool can be seamlessly integrated with other *in silico* tools commonly used for computer-aided synthesis planning. For example, ‘Fast Filter’ was used in this study to evaluate the likelihood the reaction will proceed in the forward direction.^18^ This binary classifier was previously reported to have reasonable performance at this task with a false positive rate of 1.5%.^18^ Therefore, reactions not likely to work for a given molecule are unlikely to pass through the filter. On the other hand, other forward predictors with improved performance have been reported in the literature.^25,26^ Further, machine learning models can predict the conditions that could be used to run a reaction.^27,28^ The diversification reactions proposed by our algorithm also has the potential to be integrated with some of these other tools, in a fashion similar to Fast Filter, to improve the quality of proposed suggestions. However, such integration would require additional compute resources.

Our approach plans synthetic routes for novel molecules as it generates them. It employs reaction rules defined by a large corpus of reactions to generate analogs. This approach contrasts the one employed by deep generative models. These models are trained on a database of molecules to learn the underlying distribution of chemical space, which enables them to propose novel molecules that are similar to the ones in the training database but not identical. Deep generative models are not explicitly designed to consider synthesizability of novel molecules.^4^ The present approach implicitly considers synthesizability-an important practical consideration in molecular discovery programs.

Our method adds to the capabilities of other generative models that consider synthesizability.^5,6^ First, it can diversify the input molecule using a wider range of reaction chemistries reported in the patent literature and routinely employed by synthetic chemists. Second, by using late-stage reactive intermediates for diversification, chemists do not have to resort to *de novo* synthesis for obtaining analogs proposed by the algorithm. As a result, structure–activity relationship studies designed using this algorithm could be less labor intensive because a majority of the synthesis plan until the reactive intermediate will be conserved.

The algorithm takes a holistic approach to diversification by identifying suitable reactive sites within a molecule and proposing chemically reasonable chemistries for each site. By using similarity for information retrieval from the database and ranking proposed diversification suggestions, the algorithm considers the impact of functional groups distant from the reactive site on the proposed reaction. Both these aspects add to the existing capabilities of algorithmic enumeration using reaction templates.^7,8^ By considering context beyond the sites present in the reaction template, this similarity-based approach increases the likelihood that proposed suggestions are chemically reasonable. Moreover, by considering additional reactive sites in the target beyond the substructure present in the template, our approach modifies the compound at different locations to increase the chemical diversity of the proposed suggestions.

The algorithm, intentionally, operates within the scope of chemistry known in the dataset and described in Table 1. It applies reaction templates and building blocks commonly used in the patent literature to decorate an input scaffold. The algorithm can, in principle, be used with proprietary reaction datasets to bias suggestions towards reaction chemistries and building blocks readily available within an organization. Unlike generative models, this approach does not generate novel scaffolds. Suggestions that are made outside the scope of the chemistry present in the dataset are likely to be uncertain.

## 5. Conclusion

We have developed a synthesis planning algorithm for structure–activity relationship studies. It leverages a large corpus of reaction data from the U.S. patent literature to suggest transformations for changing the chemical structure of a molecule. By recursively applying the algorithm, it was possible to generate a large virtual library of analogs. The tool's algorithms and dataset are open access. It can be used by chemists for brainstorming suggestions for structure–activity relationship studies. Further, it can also be used to generate virtual libraries of synthesizable analogs for *in silico* screening. Lastly, this work sets the stage for further development of synthesis planning tools for structure–activity relationship studies by incorporating property considerations or enzymatic suggestions.

Data-science based approaches have been repurposed to employ reaction strategies from different sub-fields of chemistry by simply changing the underlying dataset, to a first approximation. For example, similarity based organic retrosynthesis algorithm has been repurposed to perform enzymatic retrosynthesis.^11^ The present diversification algorithm, which employs organic chemistry, could serve as the foundation for computational approaches that propose enzymatic late-stage diversification strategies. Enzymes are selective catalysts; their selectivity can also be further tuned using protein engineering techniques. Enzymatic reactions also occur in mild reaction conditions; as a result, the probability of unwanted side reactions is reduced. These properties of enzymes make them well suited for application in late-stage diversification.^29^ A computational tool could help chemists, whose formal training differs from that of enzymologists, identify enzymatic opportunities for late-stage diversification.

The problem formulation strategy used in this study sets the stage for further exploration of different retrosynthesis algorithms for synthesis planning of structure–activity relationship studies. For example, multi-step retrosynthesis algorithms have been designed to prioritize discovery of pathways that start from simple buyable compounds, a desired property of retrosynthesis plans.^18^ The goal of structure–activity relationship studies in molecular discovery programs is the identify compounds with desired properties, such as efficacious and safe pharmaceutical agents. By employing mature algorithms developed for retrosynthesis,^18^ multi-step enumeration algorithms could be designed to selectively propose pathways that ultimately result in molecules likely to possess the desired properties.

## Data availability

The tool's algorithms are available at https://github.com/karthiksankar93/CompoundDiversification. FastFilter is publicly available at https://askcos.mit.edu/. Additional information on the supporting tables and figures are provided in the ESI.†

## Author contributions

KS- conceptualization, data curation, investigation, methodology, writing – original draft preparation. KFJ- conceptualization, methodology, writing – review & editing, supervision, funding acquisition.

## Conflicts of interest

There are no conflicts to declare.

## Supplementary Material

SC-015-D4SC00523F-s001
