# Supplementary material for: Similarity based functionalization for enumeration of synthetically plausible chemical libraries surrounding a target
Source: Chem Sci. 2024 May 24;15(26):10221–31. doi: 10.1039/d4sc00523f (PMC11220589; doi:10.1039/d4sc00523f)
Supplement: SC-015-D4SC00523F-s001 [file SC-015-D4SC00523F-s001.pdf]

Electronic Supplementary Material (ESI) for Chemical Science.
This journal is © The Royal Society of Chemistry 2024

SUPPORTING INFORMATION AND FIGURES

Similarity based functionalization for enumeration of synthetically plausible chemical libraries surrounding a target

Karthik Sankaranarayanan[a,b,*] and Klavs F. Jensen[b,*]
a Department of Agriculture and Biological Engineering, Purdue University, West Lafayette, Indiana 47907
b Department of Chemical Engineering, Massachusetts Institute of Technology; 77 Massachusetts Avenue, Cambridge, Massachusetts 02139, United States.

Table of Contents:

Supporting tables and figures 2
- Figure S1: Recorded reactions associated with compounds (6)-(10). 2
- Figure S2: Different fingerprint settings and similarity metrics for one-step enumeration are evaluated using the validation dataset. 3
- Figure S3: Randomly selected example from the test set (Example 1). 4
- Figure S4: Randomly selected example from the test set (Example 2). 5
- Figure S5: Randomly selected example from the test set (Example 3). 6
- Figure S6: Randomly selected example from the test set (Example 4). 7
- Figure S7: Randomly selected example from the test set (Example 5). 8
- Figure S8: Example diversification schemes from the literature. 9
- Table S1: Time taken, and number of analogs generated for every iteration. 10

Methods 11
- Enumeration algorithm evaluation: Top-k accuracy analysis 11
- Template extraction 12

Buyability of co-reactants 14
- Method 14
- Result 14

Top-N Accuracy: Chemical sensibility analysis using a graph-convolutional neural network model 15
- Method 15
- Result 15

Filtering analogs using a property constraint 16

References 17

Supporting tables and figures

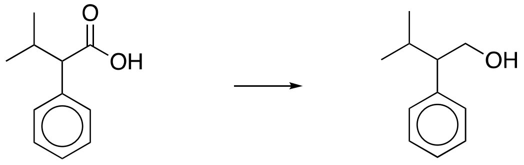

(6)

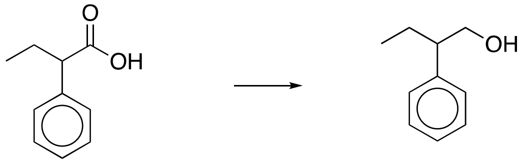

(7)

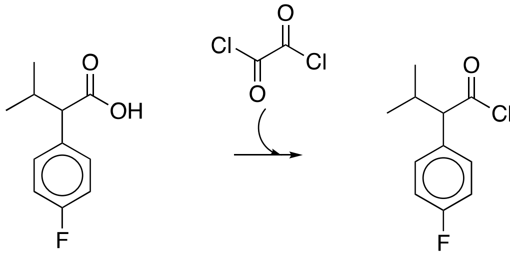

(8)

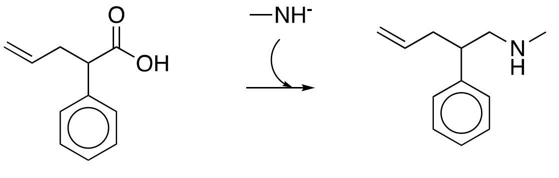

(9)

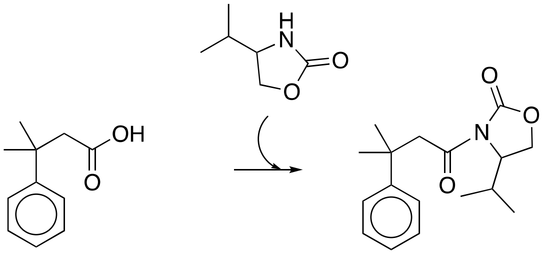

(10)

Figure S1: Recorded reactions associated with compounds (6)-(10). (6) is the test set molecule, and **(7)-(10)** are precedent molecules in the knowledgebase chemically similar to **(6)**. The approach hypothesizes that reactions associated with **(7)** - **(10)** are likely applicable to **(6)**. Applying the reaction template associated with **(7)** to the test set molecule **(6)** will recover the recorded product.

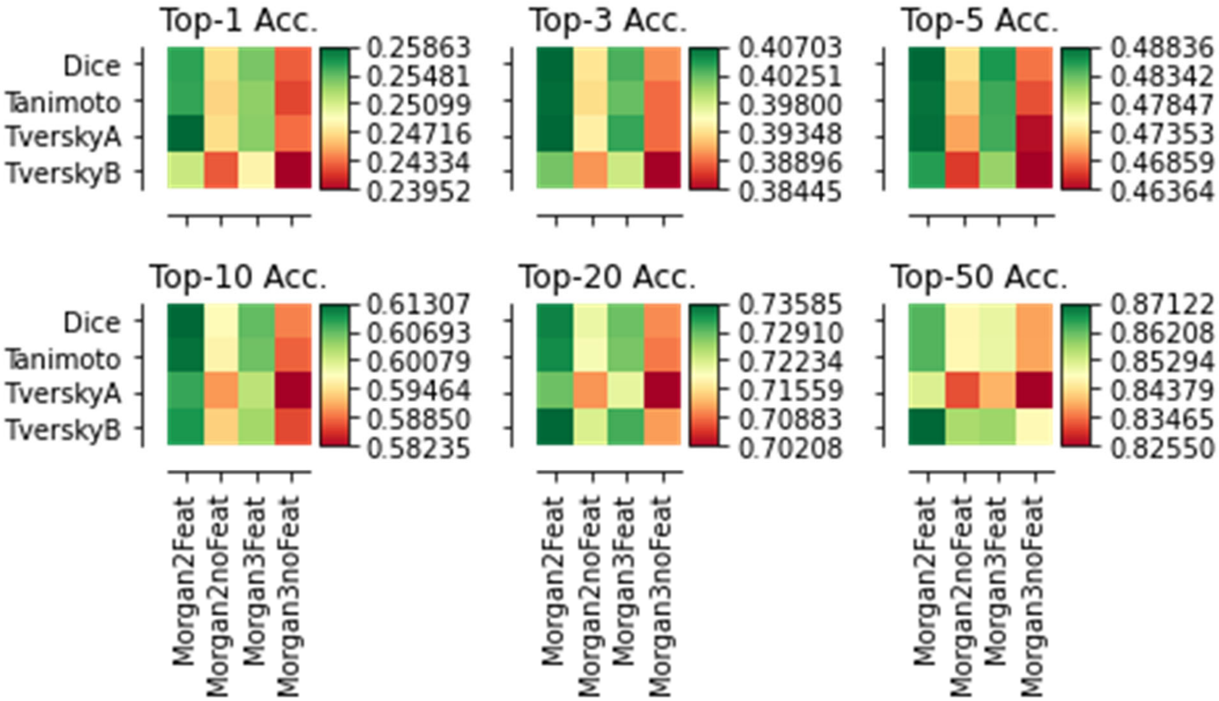

Figure S2: Different fingerprint settings and similarity metrics for one-step enumeration are evaluated using the validation dataset. 'TverskyA' and 'TverskyB' are Tversky similarity metrics with (α=1.5, β=1.0) and (α=1.0, β=1.5), respectively (see equation 3). 'Morgan2Feat' and 'Morgan2noFeat' refer to Morgan fingerprints of radius =2 with and without features, respectively. 'Morgan3Feat' and 'Morgan3noFeat' refer to Morgan fingerprints of radius =3 with and without features, respectively. The top-N accuracy is not a strong function of the fingerprint settings and similarity metrics tested. As a result, the Morgan2Feat fingerprint and Tanimoto similarity metric were used for this study.

Recorded reaction (85785)

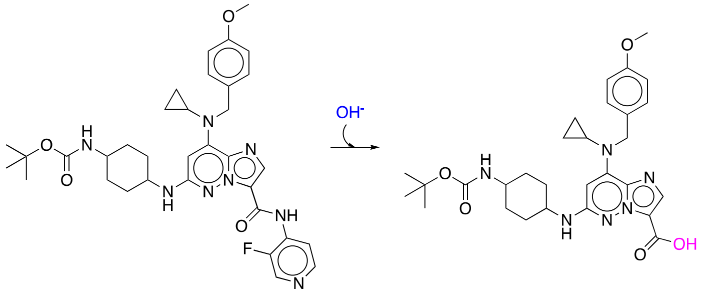

Top recommended co-reactants (in blue) and products (in black):

Rank 1:

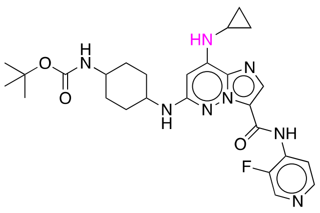

0.46

Rank 2:

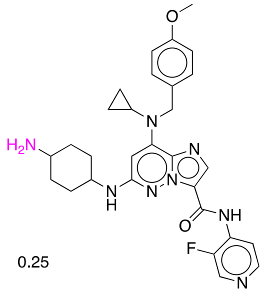

0.25

Rank 3:

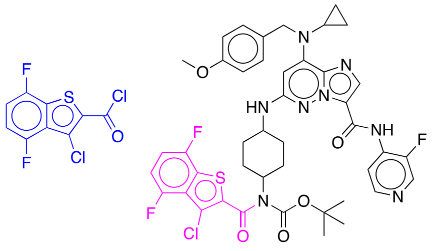

0.24

Rank 4:

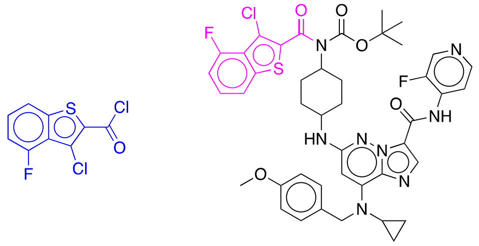

0.24

Rank 5:

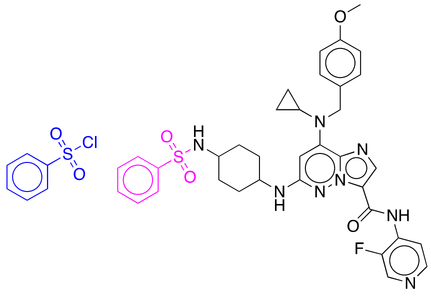

0.21

Rank 6:

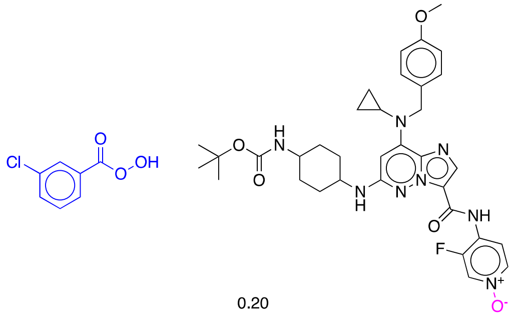

0.20

Figure S3: Randomly selected example from the test set (Example 1). For every proposed reaction, the co-reactant is shown in blue. The product is shown in black and any structural change resulting from the reaction is highlighted in pink. The rank and overall similarity score are labeled above and below every suggestion, respectively. The similarity-based approach is unable to find an analog of the recorded product.

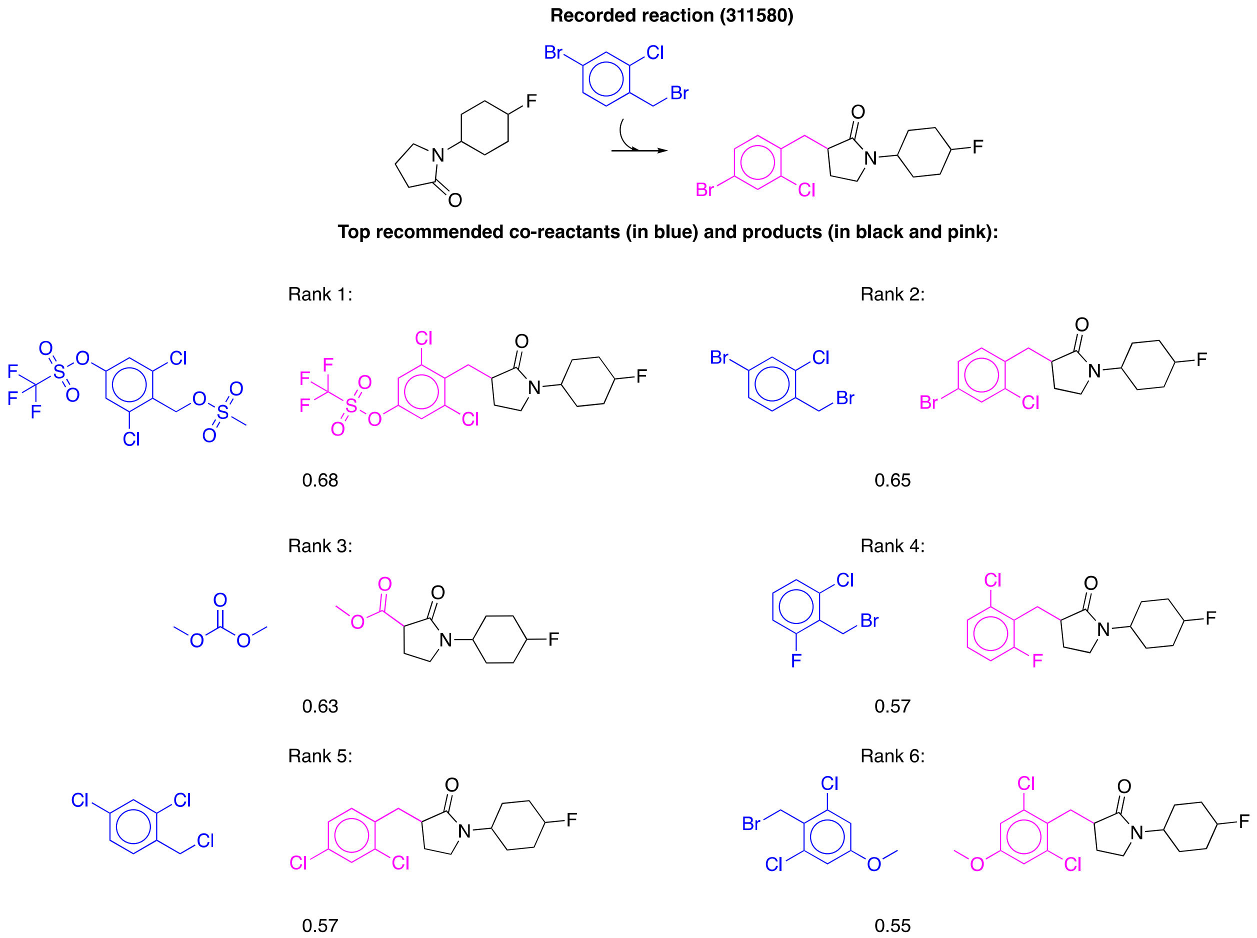

Figure S4: Randomly selected example from the test set (Example 2). For every proposed reaction, the co-reactant is shown in blue. The product is shown in black and any structural change resulting from the reaction is highlighted in pink. The rank and overall similarity score are labeled above and below every suggestion, respectively. The similarity-based approach proposes an analog of the recorded product with rank 2.

Recorded reaction (321538)

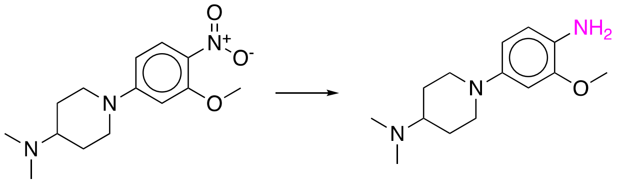

Top recommended co-reactants (in blue) and products (in black and pink):

Rank 1:

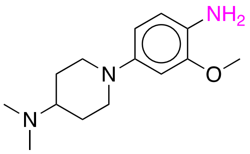

0.61

Rank 2:

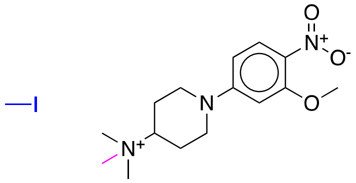

0.46

Rank 3:

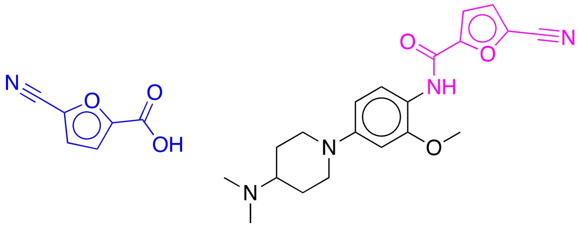

0.43

Rank 4:

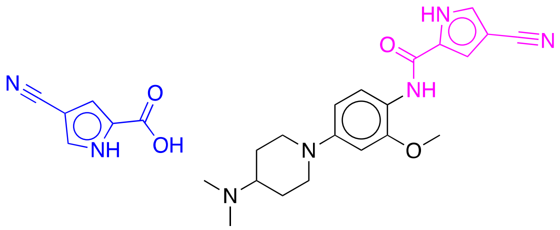

0.41

Figure S5: Randomly selected example from the test set (Example 3). For every proposed reaction, the co-reactant is shown in blue. The product is shown in black and any structural change resulting from the reaction is highlighted in pink. The rank and overall similarity score are labeled above and below every suggestion, respectively. The similarity-based approach proposes the recorded product with rank 1.

Recorded reaction (386835)

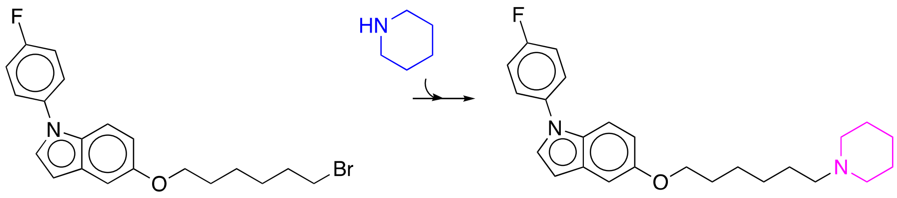

Top recommended co-reactants (in blue) and products (in black and pink):

Rank 1:

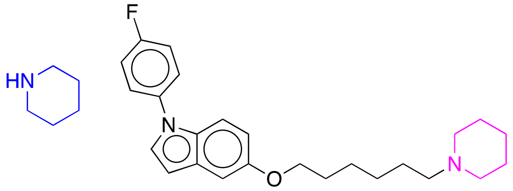

0.93

Rank 2:

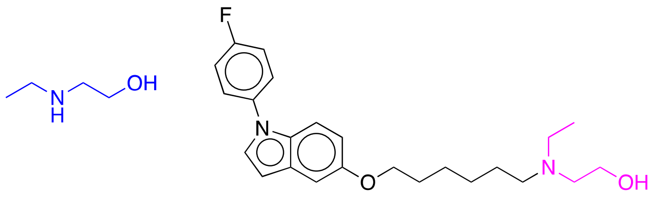

0.77

Rank 3:

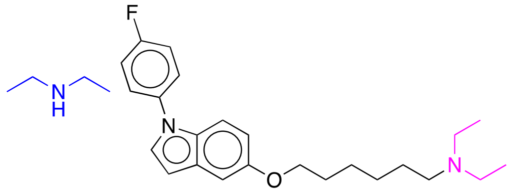

0.76

Rank 4:

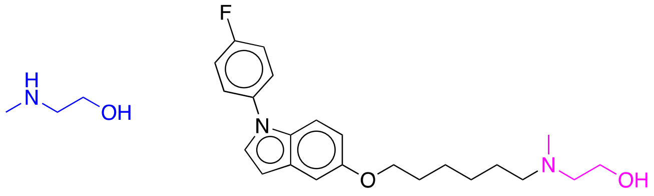

0.64

Rank 5:

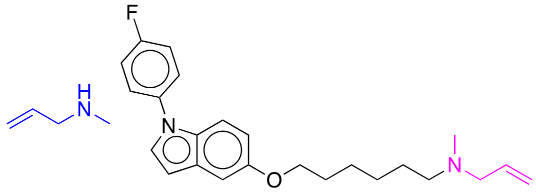

0.49

Rank 6:

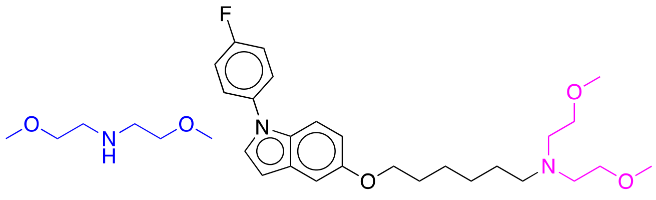

0.43

Figure S6: Randomly selected example from the test set (Example 4). For every proposed reaction, the co-reactant is shown in blue. The product is shown in black and any structural change resulting from the reaction is highlighted in pink. The rank and overall similarity score are labeled above and below every suggestion, respectively. The similarity-based approach proposes the recorded product with rank 1.

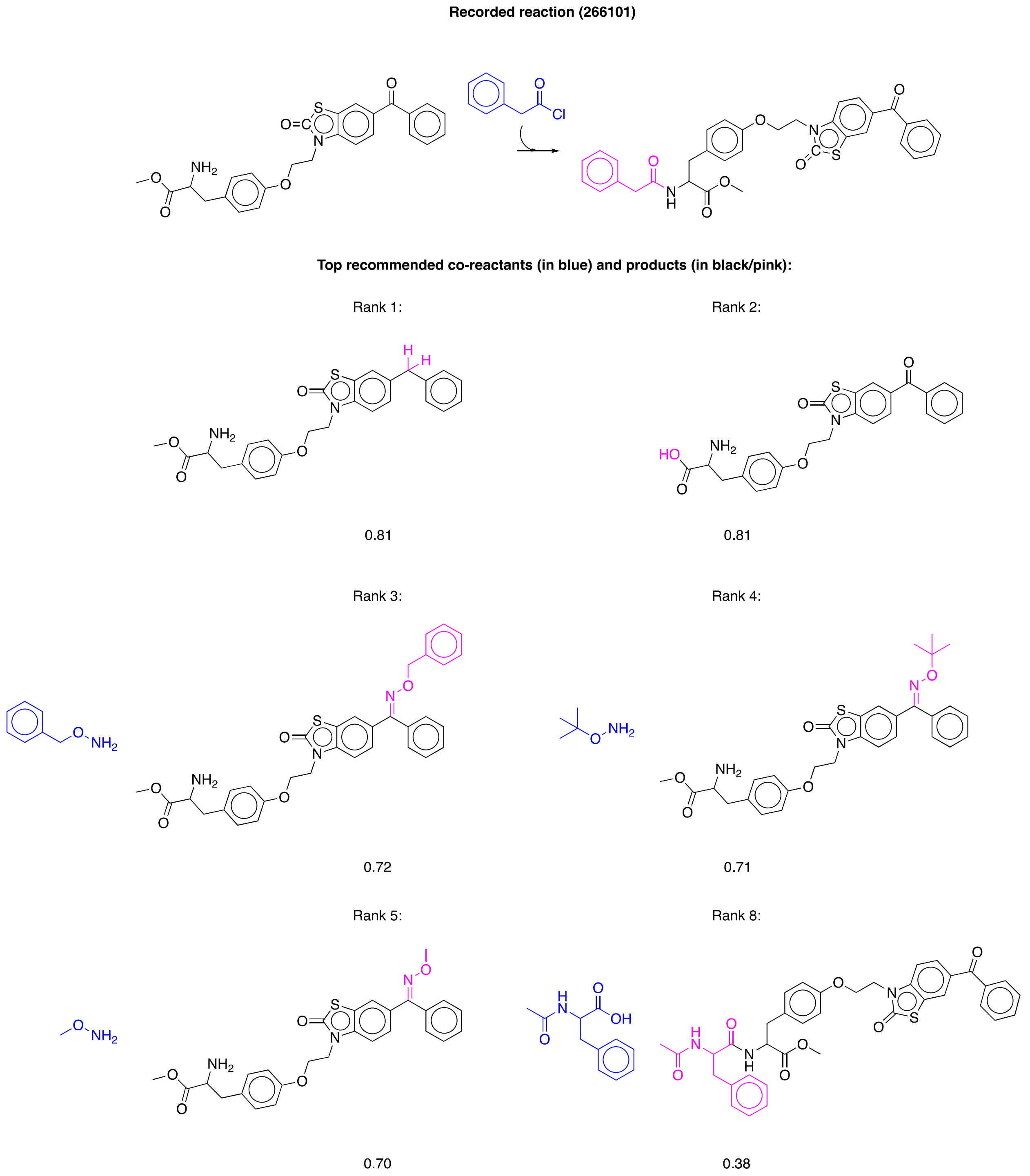

Figure S7: Randomly selected example from the test set (Example 5). For every proposed reaction, the co-reactant is shown in blue. The product is shown in black and any structural change resulting from the reaction is highlighted in pink. The rank and overall similarity score are labeled above and below every suggestion, respectively. The similarity-based approach proposes a close analog of the recorded product with rank 8.

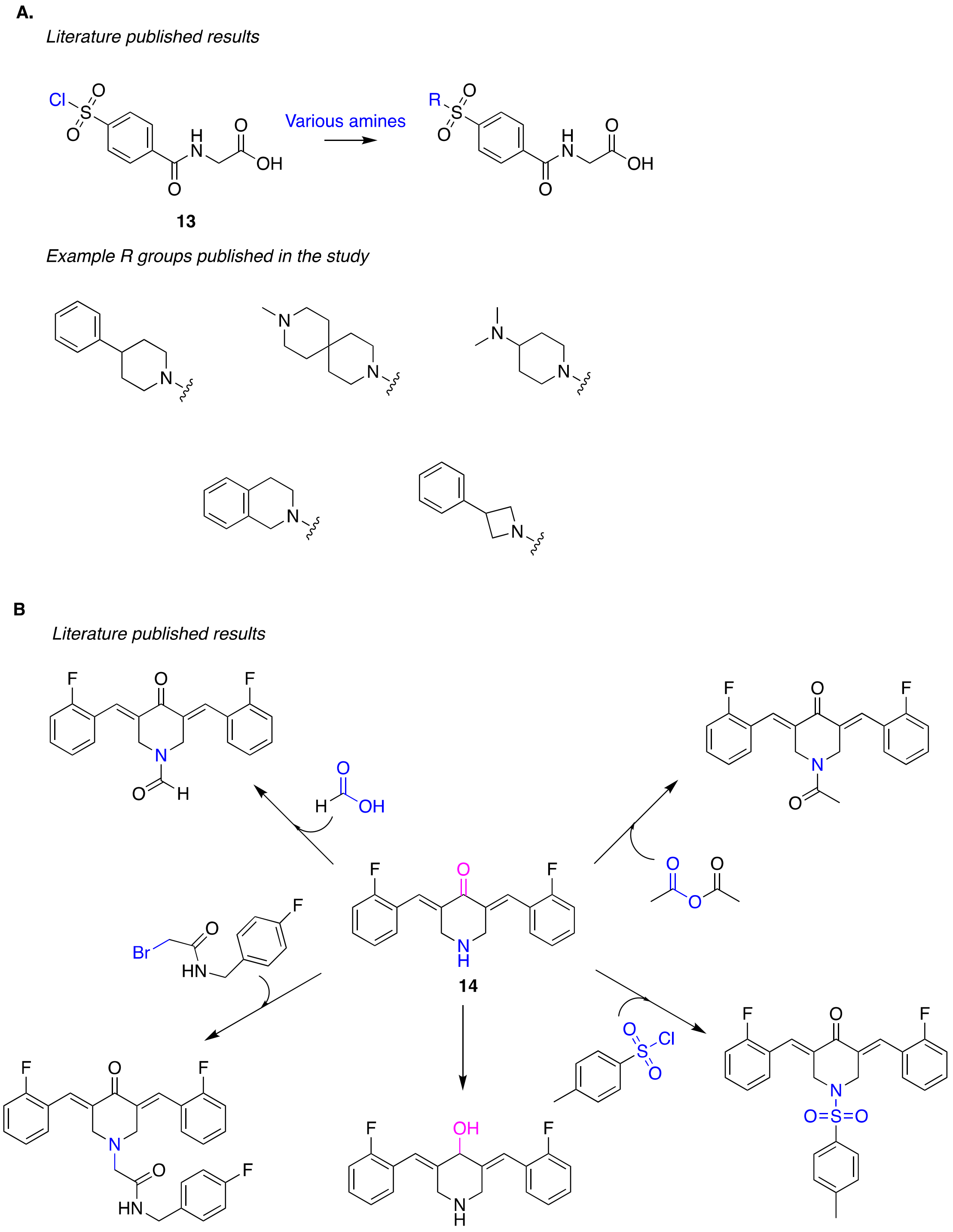

Figure S8: Example diversification schemes from the literature. (associated with Figure 6) (A) A selected reaction scheme for lead compound diversification by Mann and co-authors. Reactive intermediate **13** was treated with amines to obtain N-substituted-sulfonamides. Exemplary analogs generated in the study are described here. (B) A selected reaction scheme for lead compound diversification by Lagisetty and co-authors. Reaction intermediate **14** was diversified using different chemistries at different sites (marked with different colors). Each reaction described here is likely to contain its own distinct SMARTS pattern.

Table S1: Time taken, and number of analogs generated for every iteration.

Iteration	Number of analogs	Time taken
1	28	1.3s[#]
2	410	2.7 min[#]
3	3,566	2.05 min
4	27,725	7.25 min
5	275,718	35 min
6	2,540,954	5 hr

[#] These iterations were not parallelized, and computations were performed on a single core.

Methods

Enumeration algorithm evaluation: Top-k accuracy analysis

Here, we restate our approach to performing the top-k accuracy analysis in greater detail.

Data Processing:

USPTO 500k dataset previously published by Jin *et al.* was used for this study.[1] The chemical reactions in this dataset often have multiple reactants; we process the dataset so that each multi-reactant reaction is converted into a single-reactant '*pseudo*' reaction. This pseudo reaction comprises the most complex reactant and the corresponding reaction products; the other less complex reactants were removed to facilitate this approach. Molecular complexity was evaluated using SCScore. This procedure is described in the following algorithm:

1. For every reaction SMILES, individual reactant SMILES was extracted.
2. If the reactant contributes atoms towards the product molecule(s), the SCScore of the molecules was computed. Reactants that do not contribute atoms towards the product molecule(s) were not further considered in this analysis.
3. The reactant molecule with the highest SCScore was identified.
4. Then, a pseudo reaction SMILES was constructed. This pseudo reaction comprises the reactant with the highest SCScore and products that contain at least one atom originating from this reactant.

Reaction templates were extracted, and their quality was verified computationally. Only reactions with high quality templates were used in this study, and other reactions were filtered out from the dataset. This procedure is described in the following algorithm:

1. For every pseudo reaction, a reaction template that describes the underlying transformation in a generalized fashion is extracted. Template extraction procedure and minor changes to RDChiral[2] are described separately in this Supporting Information (See section titled 'Template Extraction').
2. The extracted template is applied to the reactant. If the recorded reaction products were successfully recovered, this pseudo reaction was used in the study. Otherwise, the pseudo reaction was filtered out and not used in this study.

We canonicalize the reaction SMILES string to remove duplicate transformations. Then, we split the dataset randomly into training (80%), validation (10%), and test (10%) splits using the reactant SMILES string. There were *ca.* 348196, 43525, 43525 reactants in the training, validation, and test splits, respectively.

Enumeration Approach:

1. Calculate the Morgan fingerprint (radius =2, using features) of the target compound.
2. Calculate a reactant Tanimoto similarity score, s_{reac}, between the target compound and each reactant that appears in the training set.

3. Iterate through each of the precedent reactions from the knowledge base in order of decreasing reactant similarity. For computational efficiency, this considers the 100 most similar reactants only. For each of these reaction precedents, extract a localized reaction template based on the atom mapped transformation, using RDChiral (modified).
4. Still iterating through the precedent reactions, apply the extracted template to the target molecule to get candidate products.
5. For each candidate product generated in the previous step, compute the candidate product's Morgan fingerprint. Then, compare it to the reaction precedent's products' fingerprint to get a second similarity score, s_{prod}. This score reflects how similar the products of the known reaction are to the proposed products of this theoretical reaction.
6. Still for each candidate precursor set, multiply the reactant similarity score s_{reac} with the product similarity score s_{prod} to get the overall similarity $s = s_{prod} \cdot s_{reac}$. This score represents the extent to which the proposed enumeration reaction is analogous to the precedent reaction.
7. Rank all enumerated products by their overall scores, s. Remove any duplicates in the candidate product list as determined by their isomeric SMILES string, while retaining only the highest score when there are multiple entries.

Top-k accuracy evaluation

1. Calculate the Morgan fingerprint (radius =2, using features) of the recorded reactant and product.
2. Calculate the baseline Tanimoto similarity score, $s_{baseline}$, between the recorded reactant and product
3. Iterate through the ranked enumerated product list in the order of increasing ranks. For each of these enumerated products, compute the enumerated product's Morgan fingerprint. Then, compare it to the recorded product's fingerprint to get the enumerated product Tanimoto similarity score, $s_{enumprod}$.
4. If the baseline similarity score $s_{baseline}$ is greater than or equal to the highest enumerated product similarity score $s_{enumprod}$, then no solution was found. Otherwise, the rank associated with highest $s_{enumprod}$ score was recorded.

Template extraction

RDChiral was used for template extraction and application.[2] By design, the problem was formulated as an inverse of the retrosynthesis problem. This allowed us to take advantage of the techniques that have been developed for retrosynthetic template extraction and application. The role of reactants and products of the pseudo reaction were reversed during the retrosynthetic template extraction. That is to say, the reactants of the pseudo reaction were fed as products to RDChiral and vice versa. This approach enabled us to employ RDChiral with minimal modifications.

One minor change was made to RDChiral to ensure successful template extraction and application. The 'MAXIMUM_NUMBER_UNMAPPED_PRODUCT_ATOMS' parameter was changed from the default setting of '5' to '5000'. This was necessary because the original reactions from the USPTO-500k dataset do not contain information about by-

products. This minor setting change ensured that we were able to extract templates from reactions containing reactants that contributed more than 5 atoms towards by-product formation.

Buyability of co-reactants

For a reaction in the dataset with multiple reactants, we constructed a corresponding *pseudo* reaction that contained the most complex reactant and the original products; the other less complex reactants were removed from the reaction (henceforth, these removed reactants are referred to as ‘coreactants’). These co-reactants can be likened to building blocks. Here, we evaluable the commercial availability of these compounds.

Method

For every reaction, co-reactants were searched using the ASKCOS buyable database.[3,4] If all co-reactants associated with a given reaction are listed as commercially available in the ASKCOS buyable database, then the reaction is classified as having co-reactants that are buyable. If at least one co-reactant is not a buyable compound on ASKCOS, then the reaction is classified as having co-reactants that are not buyable. These co-reactants would have to be synthesized or searched using other buyable compound databases.

For co-reactants that were not listed on ASKCOS as buyable, we randomly sampled ten compounds and used to ASKCOS tree-search algorithm to evaluate synthesizability.[3,5]

Result

There are a total of 435,246 reactions in the dataset. 382,086 reactions (~88% of the dataset) had co-reactants that were listed as buyable in the ASKCOS buyable database or simply did not need any co-reactants. Further, nine of the ten randomly sampled non-buyable compounds had many ASKCOS predicted synthesis pathway (57-200 trees).

Top-N Accuracy: Chemical sensibility analysis using a graph-convolutional neural network model

To complement the analysis performed using the fast-filter predictor, we also employed the graph convolutional neural network model trained by Coley and co-authors.[6] This serves as a second approach to understand the chemical sensibility of the reactions considered successful in the Top-N accuracy analysis.

In the test set comprising 44k reactions, roughly 90% (38,738 reactions) of the cases were able to recover recorded products or close analogs, and these cases considered to be successful are further analyzed. For 22,233 reactions, our algorithm recovered the exact reaction recorded on the test set. This was determined by an exact SMILES string match between the algorithm proposed reaction and the recorded reaction in the test set (*i.e.,* from the U.S. patent literature). The remaining 16,505 reactions were further analyzed using the trained graph convolutional neural network model.

Method

First, any duplicate reactions were removed. There was a total of 12804 unique reactions in the 16505 total reactions. We used a graph convolutional neural network model trained by Coley and co-authors and currently implemented on askcos.mit.edu.[6,7] The settings employed for the forward predictor were 'wldn5', and 'uspto_500K'. These model settings match closely with the settings used in the original publication. Performance was measured using top-N accuracy for N = {1,3,5,10,20,50}; this is defined as the fraction of the 12804 reactions where the similarity algorithm suggested product is predicted to be chemically sensible by the trained graph convolutional neural network model with rank $\leq$ N.

Result

Table S2: The trained graph convolutional neural network model ranks the 12804 reactions proposed by our algorithm highly, indicating chemical sensibility of the proposed transformations.

**Top-*n***	**Accuracy (%)**
1	77
3	85
5	88
10	91
20	93
50	94

Filtering analogs using a property constraint

The library of analogs generated by recursive application of similarity-based enumeration in Figure 7 were evaluated using the 'QED' property filter.[8] 'QED' is a property that was originally described in 'Quantifying the chemical beauty of drugs' by Bickerton and co-authors.[8] QED evaluates the 'drug-likeness' of a molecule based on an analysis of observed distribution of physical-chemical properties of approved drugs. QED scores range from 0 to 1 (higher scores are more drug-like). QED is a model property for illustrative purposes *only.*

The QED score implementation in RDKit was used for this analysis.[9] The QED score for all ~2.5 Million analogs was calculated. The analogs were filtered to identify molecules with QED scores greater than the input molecule (QED_{input} = 0.80235). A selection of molecules with improved QED scores are shown in Figure S9.

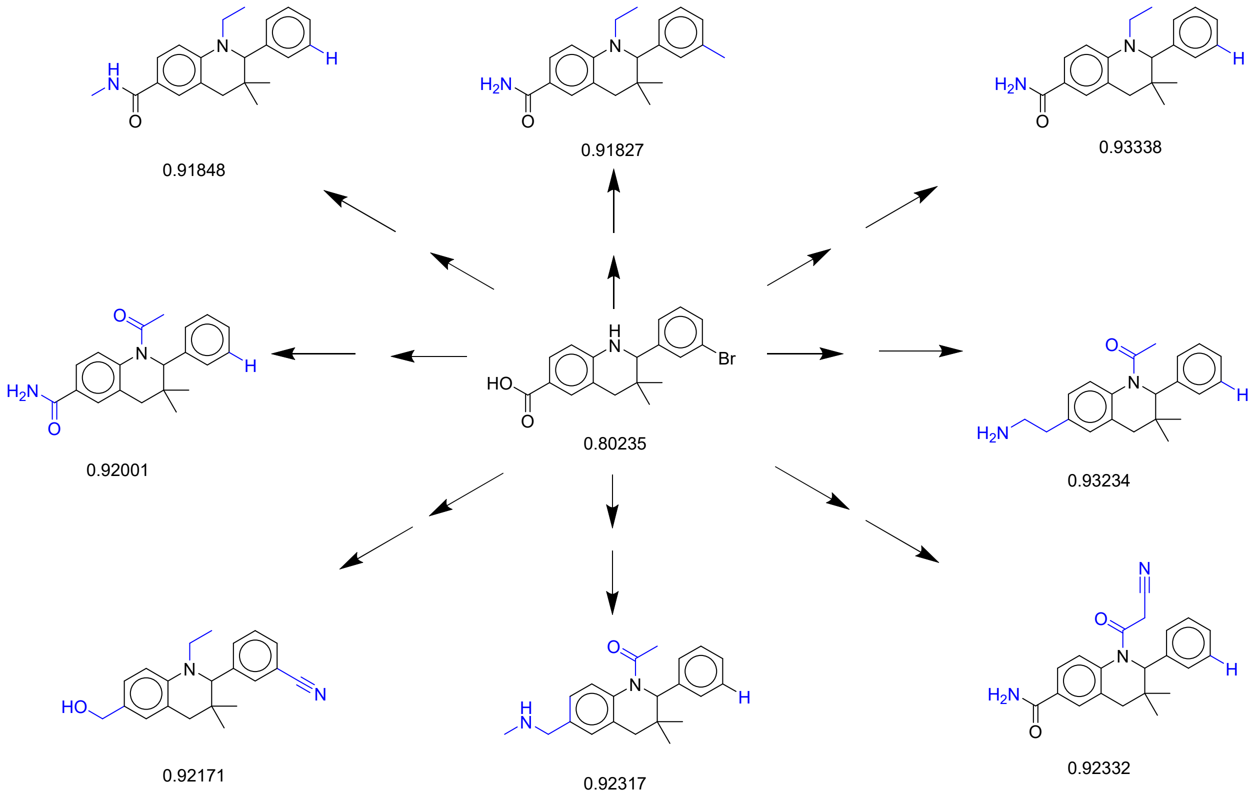

Figure S9: Analogs with improved QED property were identified using our algorithm and a 'QED' property prediction algorithm. Core structure is in black, and proposed structural modifications are in blue.

References

(1) Jin, W.; Coley, C. W.; Barzilay, R.; Jaakkola, T. Predicting Organic Reaction Outcomes with Weisfeiler-Lehman Network. *arXiv:1709.04555 [cs, stat]* **2017**.

(2) Coley, C. W.; Green, W. H.; Jensen, K. F. RDChiral: An RDKit Wrapper for Handling Stereochemistry in Retrosynthetic Template Extraction and Application. *J. Chem. Inf. Model.* **2019**, *59* (6), 2529–2537. https://doi.org/10.1021/acs.jcim.9b00286.

(3) Coley, C. W.; Thomas, D. A.; Lummiss, J. A. M.; Jaworski, J. N.; Breen, C. P.; Schultz, V.; Hart, T.; Fishman, J. S.; Rogers, L.; Gao, H.; Hicklin, R. W.; Plehiers, P. P.; Byington, J.; Piotti, J. S.; Green, W. H.; Hart, A. J.; Jamison, T. F.; Jensen, K. F. A Robotic Platform for Flow Synthesis of Organic Compounds Informed by AI Planning. *Science* **2019**, *365* (6453), eaax1566. https://doi.org/10.1126/science.aax1566.

(4) *ASKCOS Buyable Database*. https://askcos.mit.edu (accessed 2024-04-20).

(5) *ASKCOS Tree Builder*. https://askcos.mit.edu/network?tab=IPP (accessed 2024-04-20).

(6) Coley, C. W.; Jin, W.; Rogers, L.; Jamison, T. F.; Jaakkola, T. S.; Green, W. H.; Barzilay, R.; Jensen, K. F. A Graph-Convolutional Neural Network Model for the Prediction of Chemical Reactivity. *Chem. Sci.* **2019**, *10* (2), 370–377. https://doi.org/10.1039/C8SC04228D.

(7) *ASKCOS Forward Predictor*. https://askcos.mit.edu/forward?tab=forward (accessed 2024-04-21).

(8) Bickerton, G. R.; Paolini, G. V.; Besnard, J.; Muresan, S.; Hopkins, A. L. Quantifying the Chemical Beauty of Drugs. *Nat Chem* **2012**, *4* (2), 90–98. https://doi.org/10.1038/nchem.1243.

(9) *RDKit: Open-source cheminformatics*. https://www.rdkit.org/ (accessed 2024-04-21).
